# Associations of sport participation with subjective well-being: a study consisting of a sample of Chinese school-attending students

**DOI:** 10.3389/fpubh.2023.1199782

**Published:** 2023-06-23

**Authors:** Tianzhuo Liu, Dong Li, Hongying Yang, Xinli Chi, Jin Yan

**Affiliations:** ^1^School of Physical Education, Northeast Normal University, Changchun, Ji Lin, China; ^2^Department of International Culture Education, Chodang University, Muan, Republic of Korea; ^3^Library of Beijing Sport University, Beijing, China; ^4^School of Psychology, Shenzhen University, Shenzhen, China; ^5^Centre for Active Living and Learning, University of Newcastle, Callaghan, NSW, Australia; ^6^College of Human and Social Futures, University of Newcastle, Callaghan, NSW, Australia

**Keywords:** sport participation, well-being, school-aged children, adolescents, China

## Abstract

**Purpose:**

Past studies have illustrated that the impact of sports participation on school-attending students’ well-being plays a significant role in the life course of adolescence, which is a golden period for developing sound psychological qualities. However, the relationship between sports participation and subjective well-being is not clear, particularly in Chinese primary and middle schools. Therefore, the current study was aimed to explore the relationship between sports participation and subjective well-being in elementary and middle schools in China.

**Method:**

All involved children and adolescents were asked to conduct a self-report of their sociodemographic factors (e.g., sex, grade, and age), independence, and outcomes. The survey involved a two-stage sampling design (district school). Besides, in order to examine the relationship between participation in sports and subjective well-being, a self-report questionnaire was used. Logistic regression with 95% confidence interval and odds ratios (ORs), was conducted to investigate the relationship between sports participation and subjective well-being.

**Results:**

A total of 67,281 participants in total provided complete data for the final analysis of the current study. The percentage of boys and girls was 51.9% and 48.1%, namely. The current study found that compared with children who never participate in sports, those children who participated sports in 1–3 times a month, 1–2 times a week, and 3 times a week and above were more likely to enjoy better well-being. Compared with children who never participate in sports, those children who in every grade participated sports in 1–3 times a month, 1–2 times a week, and 3 times a week and above were more likely to achieve better well-being.

**Conclusion:**

Our current study offered the positive effect of sports participation on children and adolescents’ subjective well-being. For schools and governments, further studies are needed to focus on sports participation and positive feedback on adolescents’ mental health, and the three parties’ endeavors should be intervened.

## 1. Introduction

Sports participation refers to the attitude and behavior of students taking the initiative to participate in sports activities, it is only the overall description of sports goals (1–3). From the perspective of sociology and psychology, sports participation is an important strategy to realize children’s socialization, which is essentially a socialization process (3, 4). Sports participation means victory and the investment of mental energy, and different students’ energy into activities varies with time and goals (5). This concept covers not only the external indicators of the activity, such as heart rate and mood but also the goals of the exercise. Wicker and Frick’s (6) study showed that men and women who exercised more frequently reported better subjective health, but for women, the differences in subjective health were partly due to education, economic deprivation, and work-family burdens. Eime and other scholars (7) point out that regular physical activity helps prevent chronic diseases and reduces the risk of premature death. Similarly, Kantomaa et al. (8) have shown that high levels of physical activity and cardiorespiratory fitness are positively correlated with adolescents’ self-rated health, and other studies suggested that exercise can improve a person’s self-rated health (9, 10).

Sports participation has a strong relationship with mental health. Regular physical activity has been shown to have a positive impact on various aspects of mental health including reducing symptoms of anxiety and depression, improving mood, and increasing self-esteem (11). Sports participation helps to release endorphins, which are natural mood boosters and also provide an outlet for stress and tension (12). Furthermore, exercise can improve sleep quality, which is crucial for good mental health (13). Additionally, participating in physical activity and being part of a sports team can provide social support, which is important for mental well-being (14–16). Exercise can also serve as a distraction from negative thoughts and feelings and can provide a sense of accomplishment and purpose (7). Therefore Easterlin et al. (17) supposes that sports participation is a crucial aspect of maintaining good mental health and should be incorporated into one’s daily routine. It is important to find physical activities that are enjoyable and sustainable in the long term to ensure continued participation and mental health benefits.

Adolescence is a critical stage of the life course, which is a golden period for developing sound psychological qualities (18). However, an increasing rate of mental disorders and self-harm behaviors have been seen among adolescents (19), which greatly burden society. The mental health of young people is a global public-health challenge (20). Although most mental disorders are often detected in adulthood, they always begin during adolescence. Research on adolescents’ mental health has been historically dominated by investigating risks and vulnerabilities for mental health problems (21). In the last few decades, a positive perspective on adolescent mental health with a focus on strengths and well-being is gaining increasing popularity. As a concept closely related to positive mental health, psychological resilience has drawn significant attention from researchers in the area of adolescent mental health (22). Psychological resilience is considered a protective factor or dynamic process that stimulates individuals to remain healthy or recover swiftly in the face of adversities (23). Though there is no universal definition of psychological resilience, it was mostly defined as an ability to rise above to overcome adversity or bounce back from adversity with an outcome of adaptation and adjustment, or maintenance of good mental health (24). High levels of resilience could help individuals cope with stressful situations in a positive direction toward better outcomes (25). Previous research showed that adolescents with a high level of resilience had more desirable developmental outcomes, such as healthier dietary patterns, improved sleep quality (26, 27), better academic performance (28) and social competence (29), less depressive and anxiety symptoms (30), and less suicidal ideation (31) and suicidal behaviors (32).

Subjective well-being popularly referred to as happiness or life satisfaction is also a key aspect of positive mental health. In the past decades, the empirical literature on subjective well-being has grown enormously with the remarkable progress in positive psychology (33). Subjective well-being can be defined as being satisfied with life, experiencing long-term happiness, and having fewer negative emotions (34). As such, subjective well-being is a multi-dimensional concept, including the cognitive dimension (general life satisfaction) and the affective dimension (presence of pleasant affect and absence of unpleasant affect) (35). Many researchers hold that the subjective well-being of adolescents summarizes all aspects of their well-being, by not only serving as an indicator of their mental health but also playing a role in their physical health (36). Benefits brought by subjective well-being include good physical health, longer longevity, greater self-esteem, more satisfactory social relationship, and better creativity and cognitive performance (9, 33). Considering the importance of promoting psychological resilience and subjective well-being among adolescents, more research on this topic is needed for a better understanding of positive development in adolescence (37). One primary focus is to find out modifiable factors related to psychological resilience or subjective well-being for targeted interventions.

Since the teenage years are a crucial period to develop our relationships with others and views on the outside world, scientific research has suggested that our lifestyle and even habits can have a significant impact on the drastic changes in our psychosocial and cognitive functions (38). An abundance of research evidence has proved a strong correlation between sport participation and the overall well-being of adolescents (39). Recent research has been conducted in various countries and social contexts, delving into the specific aspects of exercise and its impact on young adults’ minds and bodies. For instance, Costigan et al. (40) intended to find the intensity of physical exercise and its influence on the mental well-being of adolescents. Their research results discovered that intense physical workout is positively related to enhanced self-reported well-being among young adults, in compliance with the contemporary physical exercise guidelines advocating for more appropriate and necessary active workouts at least three times per week. Martínez-López et al. (41), from another perspective, discovered that physical activities not only have a favorable impact on teenagers’ general well-being but also facilitate their coping mechanisms when experiencing stress and pain in puberty. Moreover, in hopes of promoting mental health and targeting useful strategies to help young adults thrive in adolescence, Pigaiani et al. (42) conducted a thorough meta-analysis among Italian adolescents, concluding that participation in physical activities can remarkably contribute to adolescents’ subjective well-being, along with other crucial factors such as gaining social and familial support. However, it is noteworthy that all of these results showed a subtle deviation based on gender differences, pointing to the need for further relevant research done in the future. Such gender-based variations were displayed in the likelihood of reporting mental state changes, etc. In a word, physical activities are key to fulfilling the psychosocial development of all adolescents.

## 2. Methods

### 2.1. Procedure and participants

In March 2021, we conducted a large-scale survey in Shenzhen, a relatively developed city in China. The targeted participants were students from local public primary and secondary schools. The survey adopted a two-stage sampling design (district school), a balanced representation of geography, economic development, and rural–urban diversity was achieved. Public schools in 11 districts in Shenzhen of China were recruited. The sampling procedure involved sampling towns, and local community districts that represented a mix of rural and urban areas. China’s education system is a 6-3-3 system, including 6 years of primary school, 3 years of junior middle school, and 3 years of senior high school. The study included all students in grades 5–6 of primary school, grades 1–2 of junior middle school, and grades 1 and 2 of senior high school in the selected schools. The survey included adolescents in Grade 5 and above (≥ 10 years old) to ensure that they were cognitively able to complete the self-report questionnaire. In addition, the survey did not include students in the third grade of junior middle school and the third grade of senior high school, because they were preparing for the high school and university entrance examinations, and the curriculum was relatively compact, which might not be able to coordinate the time to participate in such a large-scale survey collectively.

Before the investigation, all participants and their guardians were informed of the purpose and overview of the investigation. We highlighted that participation was voluntary and data would be collected and analyzed anonymously. The students who agreed to participate completed the online questionnaire (about 20 min) independently in the school computer room under the leadership of the class teacher. The questionnaire was completed on the Wenjuanxing platform, a Chinese online questionnaire platform. Before entering the formal answer page, the page of electronic informed consent was displayed first. Only students who clicked the button for consent to participate in the survey button could enter the formal answer page. The survey was approved by the Research Committee of Shenzhen University (No. 2020005) and the participating schools.The approval date was May 21, 2020.

Finally, a total of 78,428 people submitted questionnaires. After completing the preliminary data cleaning (such as deleting the data of participants whose answer time was less than 300 s), 73,323 samples from 135 schools were retained, forming the preliminary database of the survey. According to the availability of the variables required for the current study in the database, a total of 67,281 students formed the analysis sample of the current study.

### 2.2. Measurements

#### 2.2.1. Independent variable (sports participation)

The item was used to measure the frequency of sports participation: “How often have you participated in sports teams or sports clubs in the past year?” Participants reported the frequency by selecting one of the following options: never, 1–3 times per month, 1–2 times per week, and 3 or more times per week. The frequency corresponding to the option increased in turn.

#### 2.2.2. Dependent variable (subjective well-being)

Subjective well-being was measured by the Chinese version of the World Health Organization Five-Item Well-being Index (WHO-5) (43). The WHO-5 consists of 5 questions, which are answered on a 6-point scale (5 = All of the time, 4 = most of the time, 3 = more than half of the time, 2 = less half the time, 1 = sometimes, 0 = never). Item scores are summed and converted to obtain a total score ranging from 0–25, where 25 presents the best possible level of well-being. Psychometric properties of the Chinese version of WHO-5 have been validated among the Chinese population (43). In the study, the Cronbach α coefficient for WHO-5 was 0.94.

### 2.3. Covariates

The data about age, BMI, grade, gender, subjective family socioeconomic status [a measure for evaluating the ethnic of participants, and the MacArthur Scale of Subjective Social Status’s adapted version was adopted to assess it (44)], educational level of parents (i.e., master’s degree or above; bachelor’s degree; senor high school; secondary school or below), and siblings (only kid or not) were collected. In further analysis, these factors were regarded as covariates.

### 2.4. Data analyses

Some questionnaires’ data were eliminated due to unreasonable or missing values. Apart from that, 67,281 participants in total provided complete data for the final analysis of the study. In addition, the statistical analysis was conducted using STATA BE 17.0 (College Station, Texas, United States). Prior to formal analysis, the complete cases were used to address missing data. Based on the descriptive statistics, the sample characteristics were described by denoting mean (M) with standard deviations (SD) for continuous variables or frequency (n) with percentage (%) for categorical variables. Eventually, an ordered logistic regression was conducted to explore the relationship between the sports team’s participation and subjective well-being after control of the covariates (i.e., gender, BMI, age, grade, siblings, parents’ education background, and ethnicity). What’s more, results were described as odds ratios (OR), with the confidence interval (CI) being 95%. Besides, statistical significance was determined by using a prior value of p below 0.05.

## 3. Results

**Figure 1:**
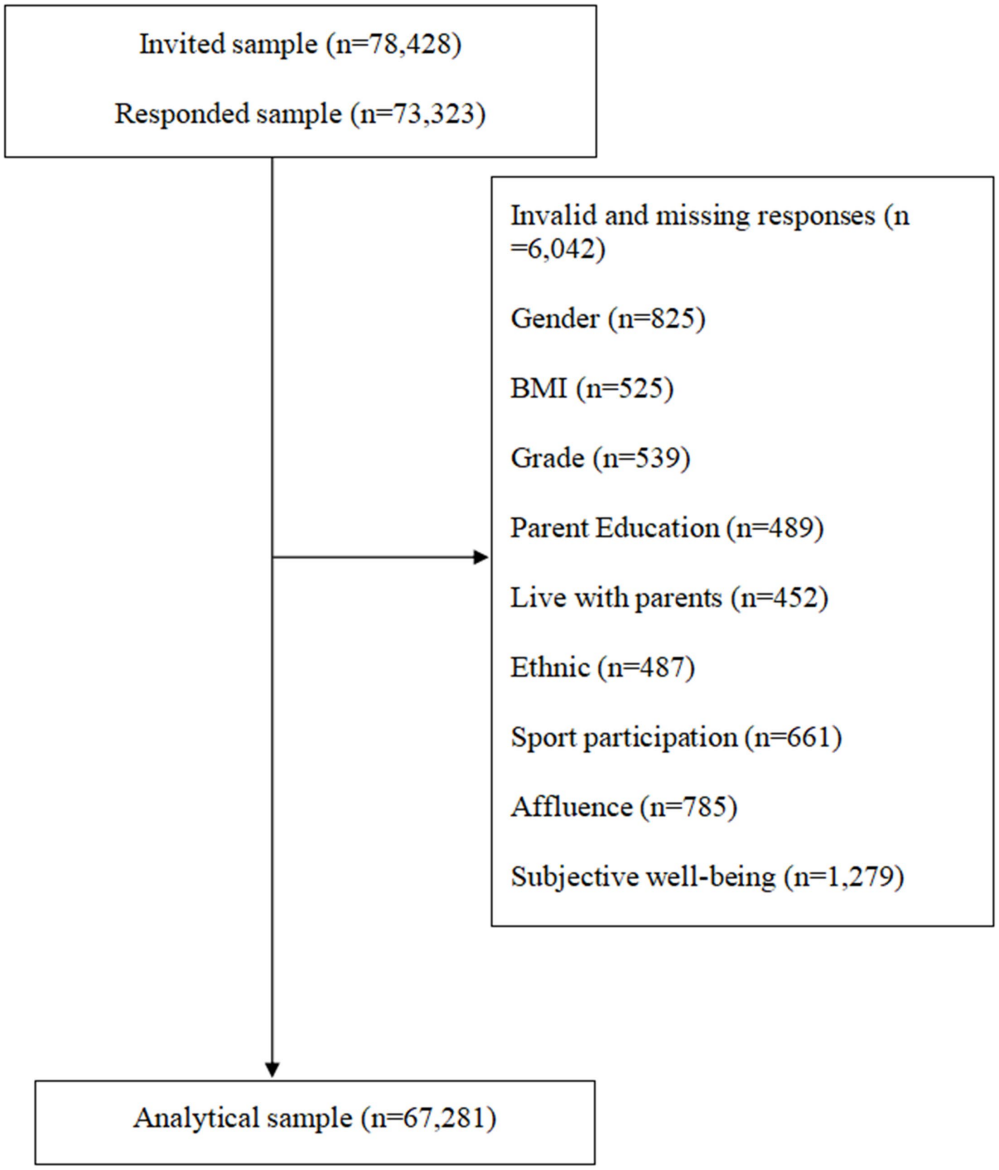
The procedure used for cleaning invalid and missing data in this study.

The demographic characteristics of the total sample have been displayed in Table 1. A total of 67,281 children (M age = 13.0 years, SD = 1.80) were involved in the final analysis (Figure 1). The percentage of boys and girls was 51.9% and 48.1%, namely. In terms of BMI, the rate of “normal” accounted for 68.1%, which was the most population, followed by the “overweight” (13.5%), and “obese” (18.4%), respectively. Regarding sport participation, around half the sample reported “Never,” and one-fifth of children reported “1–3 times/month.” More details can be found the Table 1.

**Table 1:** Demographic characteristics of the participants.

		*n*/mean	%/sd
Age		13.0	1.8
Sex			
	Boy	34,909	51.9
	Girl	32,372	48.1
BMI			
	normal	45,817	68.1
	overweight	9,051	13.5
	obese	12,413	18.4
Grade			
	Primary school	27,954	41.5
	Middle school	27,124	40.3
	High school	12,203	18.1
Father’s education level			
	Middle school and below	14,619	21.7
	High school/secondary school/vocational high school/technical school	18,159	27.0
	University/College/Higher Vocational	26,030	38.7
	Master’s/PhD	2,796	4.2
	Not clear	5,677	8.4
Mother’s education level			
	Middle school and below (including middle school)	17,617	26.2
	High school/secondary school/vocational high school/technical school	18,706	27.8
	University/College/Higher Vocational	23,922	35.6
	Master’s/PhD	1,635	2.4
	Not clear	5,401	8.0
Live with parents			
	Yes	62,836	93.4
	No	4,445	6.6
Ethnic			
	Han	65,027	96.6
	Other	2,254	3.4
Sport participation			
	Never	31,721	47.1
	1–3 times/month	15,050	22.4
	1–2 times/week	11,789	17.5
	3 times/week and above	8,721	13.0
Affluence		5.0	1.7
Subjective well-being		20.2	6.7

Overall, A positive association was found between sports participation and better well-being among Chinese children. Based on Table 2, compared with children who never engage in sports, those children who participated sports in 1–3 times a month, 1–2 times a week, and 3 times a week and above were more likely to enjoy better well-being (OR = 1.26, 95% CI: 1.22–1.30), (OR = 1.41, 95% CI: 1.36–1.46), and (OR = 1.66, 95% CI: 1.59–1.73), respectively. A similar trend has also been displayed in the boys, compared with boys who never engage in sports, those boys who participated sports in 1–3 times a month, 1–2 times a week, and 3 times a week and above were more likely to have better well-being (OR = 1.27, 95% CI: 1.21–1.33), (OR = 1.38, 95% CI: 1.31–1.46), and (OR = 1.74, 95% CI: 1.65–1.85). In terms of girls, compared with students who never engage in sports, those students who participated sports in 1–3 times a month, 1–2 times a week, and 3 times a week and above were more likely to have better well-being (OR = 1.25, 95% CI: 1.19–1.31), (OR = 1.45, 95% CI: 1.37–1.53), and (OR = 1.55, 95% CI: 1.45–1.66).

**Table 2:** Odds ratio and 95% CI for subjective well-being concerning the sex.

Overall					Boys					Girls				
Subjective well-being	OR	95% CI		Sig	Subjective well-being	OR	95% CI		Sig	Subjective well-being	OR	95% CI		Sig
Frequencies														
1–3 times a month	1.26	1.22	1.30	***p* < 0.001**	1–3 times a month	1.27	1.21	1.33	***p* < 0.001**	1–3 times a month	1.25	1.19	1.31	***p* < 0.001**
1–2 times a week	1.41	1.36	1.46	***p* < 0.001**	1–2 times a week	1.38	1.31	1.46	***p* < 0.001**	1–2 times a week	1.45	1.37	1.53	***p* < 0.001**
3 times a week and above	1.66	1.59	1.73	***p* < 0.001**	3 times a week and above	1.74	1.65	1.85	***p* < 0.001**	3 times a week and above	1.55	1.45	1.66	***p* < 0.001**

**Reference group**: never participate in sports. All models controlled for sex, age, BMI, grade, subjective family socioeconomic status, parents’ educational background, and ethnic; statistically significant at *p* < 0.05.

The associations between sport participation and grade were demonstrated in Table 3. It can be observed the similar trend in all groups of elementary, middle, and high school. For example, in terms of elementary, compared with children who never engage in sports, those children who in every grade engage sports in 1–3 times a month, 1–2 times a week, and 3 times a week and above were more likely to have better well-being (OR = 1.19, 95% CI: 1.31–1.26), (OR = 1.35, 95% CI: 1.27–1.42), and (OR = 1.62, 95% CI: 1.52–1.72). Detailed information can be found in Table 3.

**Table 3:** Odds ratio and 95% CI for subjective well-being concerning the grade.

Elementary school					Middle school					High school				
Subjective well-being	OR	95% CI		Sig	Subjective well-being	OR	95% CI		Sig	Subjective well-being	OR	95% CI		Sig
Frequencies														
1–3 times a month	1.19	1.13	1.26	***p* < 0.001**	1–3 times a month	1.30	1.24	1.37	***p* < 0.001**	1–3 times a month	1.32	1.22	1.42	***p* < 0.001**
1–2 times a week	1.35	1.27	1.42	***p* < 0.001**	1–2 times a week	1.43	1.35	1.52	***p* < 0.001**	1–2 times a week	1.54	1.41	1.68	***p* < 0.001**
3 times a week and above	1.62	1.52	1.72	***p* < 0.001**	3 times a week and above	1.66	1.55	1.77	***p* < 0.001**	3 times a week and above	1.81	1.60	2.04	***p* < 0.001**

**Reference group**: never participate in sports. All models controlled for sex, age, BMI, grade, subjective family socioeconomic status, parents’ educational background, and ethnic; statistically significant at *p* < 0.05.

## 4. Discussion

The purpose of our study was to explore the relationship between sports participation and subjective well-being. Through analysis, we can know that exercise has a very high significance in improving happiness and is also the most obvious compared with other variables. Physical exercise can improve people’s psychological emotions, self-confidence, and overall happiness, so it is beneficial to participate in physical exercise more (45). The results show similarity with the research results of western countries. Ruseski et al. (46) conducted a study on the population living in Rheinberg, Germany in 2009, and the results indicate that people who participated in sports had higher subjective well-being. The report also shows that men are happier than women generally. Mansfield and his colleagues (47) have systematically reviewed 6,587 published articles and further distinguished the influence of different sports based on functionality. Research shows that group activities and collective sports involving partners, including dance, significantly improve subjective well-being compared to meditative exercises and sports therapy (47). However, negative emotions exist in competitive sports activities, which may affect subjective well-being to a certain extent. The possible explanation is that competitive sports require participants to reach the limits of their functions, thus causing discomfort and exclusion feelings (48).

The current results displayed a positive correlation between the frequency of physical exercise and subjective well-being. Moreover, with the increase of age, the effect of exercise on well-being is more obvious, results showed that old people (51–75 years) and middle-aged people (31–50 years) were more likely to report physical benefits than young people (18–30 years) (*p* < 0.05), respectively. (49). It can be said that sports participation brings more benefits to older students, which is consistent our finding. Some studies have shown that sports can improve students’ cognition, especially those with higher requirements for physical coordination (50, 51). From the perspective of neuroscience, the development of the brain has a positive relationship with the size of cognitive ability (52, 53). It can also be considered that the more nerve fibers, dendrites, and synapses in the human brain, the stronger the cognitive ability (54). In the process of children’s growth, sports can significantly increase the number of nerve fibers, dendrites, and synapses in the brain, promoting the development of the brain and improving children’s cognitive ability (55). In terms of gender differences, the current study shows that boys generally enjoy a stronger sense of well-being than women as the frequency of sports participation goes higher (*p* < 0.01), which is consistent with previous studies (7, 46). The potential explanation lies in the sports preference difference between men and women. Men generally choose high-intensity activities like basketball, volleyball, football, and other sports which require collective participation (56). However, women tend to choose dancing, yoga, aerobic exercise, and jogging, which can be performed individually with less collective effort (57). The sports suitable for men require more parts of the brain activities with higher emotional needs and interpersonal relations.

In terms of the influence on different sports, the research shows that all exercise interventions have increased the total score of subjective well-being (58). However, the intervention effect of sports such as aerobics and yoga is better than that of antagonistic sports, which is consistent with previous research results (59, 60). Through experimental research, Kim et al. (61) found that the total score of subjective well-being of college students performing aerobics was significantly higher than that of the basketball group (*F* = 93.34, *p* < 0.001). The reason may be that students need a lot of communication and cooperation in the process of aerobics, for example, frequent communication of body language and facial expressions is easier to create harmonious interpersonal relationships (62). This harmonious learning and sports environment enables students to correctly handle interpersonal relationships, thus improving their social adaptability, and then express themselves more confidently in daily life, making it easier to obtain positive emotions, thus having a strong sense of subjective well-being. In contrast, basketball is a skill-oriented game in the play-field. It does only require students to have high technical and tactical abilities, but also requires high cooperation between team members (63, 64). In practice or competition, physical contact between students is easy to occur, and collision is inevitable. For junior high school students with unstable emotions, it is easy to have impulses, anger, and other emotions, which require higher emotional management ability to restrain themselves and suppress their bad temper. Therefore, compared with aerobics students, who participate in basketball have less subjective well-being (65). Existing research has demonstrated a dose–response relationship between cardio exercises and depression, indicating that involvement in intensive exercises would generate desirable fitness outcomes that are more inducive to psychological health (β = −0.266, *p* < 0.001) (66). Hamer et al. conducted self-reported surveys trying to discover the optimal amount of sports participation and different types to achieve improved psychological conditions (OR 0.67, 95% CI 0.61–0.75) (67). The results exhibit a dose–response relationship, with a temperate decline in mental issues with less frequent activities.

Taking China as a research background, many studies have been conducted to explore the relationship between physical activity and mental health issues. In Hong Kong, it is found that the self-efficacy and resilience shown by teenagers when performing sports activities can have a profound impact on their psychological well-being (68). Another study revealed that adolescents with low BMI scores and high levels of lung capacity, muscle strength and flexibility report better well-being, motivation and enjoyment in Chinese background (15). Sports can not only make muscles more developed but also stimulate the brain stem and provide energy, enthusiasm, and motivation for athletes (16). Intending to reveal the relationship between physical activity and subjective well-being, for example, a study conducted a questionnaire among 723 college students in China finding that a positive relationship between life satisfaction and vigorous physical activities, indicating the significant roles of regular exercise for students (69), which is in line with western studies (70, 71).

In future research, it is essential to probe into the strategy that optimizes the sport’s effect on kids’ mental health. There is a need to explore more high-quality game-based approach longitudinal and intervention studies to improve children’s physical self-perception, intrinsic motivation, well-being, physiological and underlying psychological outcomes (72). It is noteworthy that the SAAFE (supportive, active, autonomous, fair, and enjoyable) principle offers a framework for designing and delivering exercise sessions (73). Under the guidance of self-determination theory, these kinds of principles are successfully applied to two high-intensity interval training (HIIT) studies (74, 75). Despite the aversive nature of HIIT, it is considered that vigorous physical activities may be more enjoyable to kids if their design can meet the fundamental psychological demands (i.e., relatedness, competence, and autonomy) (76). For instance, autonomy may be satisfied by offering participants selections of exercises and rest interval durations. Apart from that, participants’ competence perceptions may be enhanced by giving positive feedback. At the same time, it can be more enjoyable to perform vigorous physical activities in groups, which will meet the relatedness perception.

Admittedly, the self-report of subjective well-being has some limitations, given that it may lead to bias and social desirability. What’s more, the cross-sectional design may not be able to explain the causal relationships satisfactorily. Furthermore, one of the most important limitations of this study is undoubted that no distinction was made by a type of movement other than by type of frequency, and no data regarding the differentiating different types of sports was supported in the current study. To better understand the causal relationship between sports participation in Chinese school and subjective well-being, and improve the success rate of intervention, it is essential to make a prospective research design. Besides, there are some biases in the use of a questionnaire for collecting data. Lastly, convenience sampling has an obvious disadvantage. Specifically, it may be biased, as it is likely to be not representative of the population. Given the limitations, future research needs to be carried out to solve problems and get stronger shreds of evidence.

### 4.1. Practical implications

At the same time, this study’s findings have practical implications for future research. First of all, the families, schools, and government should focus on the sports participation’s positive feedback on the adolescents’ mental health, and the three parties’ endeavors should be intervened. In addition, the government needs to tackle with disadvantages of irrational educational structures and unbalanced sports equipment, to offer teenagers perfect conditions and hardware. Second, schools should draw up sports plans and try to stimulate students’ enthusiasm, in order to make them feel positive feedback from sports participation. For example, providing participants with choices of exercises may help to satisfy autonomy. At the same time, the participants’ competence awareness can be strengthened by offering positive feedback and encouragement. Carrying out activities in groups is conducive to meeting the perception of relatedness. Beyond that, strategies, such as making use of music, can optimize the cognitive health benefits. Lastly, parents should give active guidance and capture teenagers’ interests timely, so as to maximize the physical needs of kids. All in all, sports education targeted at youth is a long-term objective, which requires high-quality macro conditions. Meanwhile, it is essential to consider the student’s individual needs to promote their participation in sports.

## 5. Conclusion

The current study has confirmed evidence for the positive relationship between sports participation in children and adolescents’ subjective well-being. In future studies, it is essential to probe into the strategy that optimizes the sport’s effect on kids’ mental health. Beyond that, the size of the effect is likely to be optimized by paying attention to the neurobiological, behavioral, and psychosocial mechanisms. Secondly, the families, schools, and government should focus on the sports participation’s positive feedback on the adolescents’ mental health, and the three parties’ endeavors should be intervened. In addition, the government should tackle the disadvantages of irrational educational structures and unbalanced sports equipment, to offer teenagers perfect conditions and hardware. All in all, sports education targeted at youth is a long-term objective, which requires high-quality macro conditions. Meanwhile, it is essential to consider the student’s needs to promote their participation in sports.

## Data availability statement

The original contributions presented in the study are included in the article/supplementary material, further inquiries can be directed to the corresponding author.

## Ethics statement

The studies involving human participants were reviewed and approved by this study was conducted based on approval from the Research Committee of Shenzhen University (No. 2020005) and schools that participated in the survey. The approval date was May 21, 2020. Written informed consent to participate in this study was provided by the participants’ legal guardian/next of kin.

## Author contributions

TL and DL: conceptualization and methodology. TL: software and validation. HY and JY: formal analysis. TL and JY: investigation and data curation. HY and DL: resources. XC: writing—original draft preparation, visualization, and project administration. JY: writing—review and editing and supervision. All authors contributed to the article and approved the submitted version.

## Funding

Research on the Reform of School Physical Education under the background of modern information technology (Jilin Province education science “13th Five-Year Plan” subject; No.GH19428). Natural Science Foundation of Guangdong Province [grant number 2021A1515011330]; Shenzhen Education Science Planning Project [grant number cgpy21001]. Shenzhen University-Lingnan University Joint Research Programme [grant number 202202001].

## Conflict of interest

The authors declare that the research was conducted in the absence of any commercial or financial relationships that could be construed as a potential conflict of interest.

## Publisher’s note

All claims expressed in this article are solely those of the authors and do not necessarily represent those of their affiliated organizations, or those of the publisher, the editors and the reviewers. Any product that may be evaluated in this article, or claim that may be made by its manufacturer, is not guaranteed or endorsed by the publisher.
